# Improving the Solid Fuel Properties of Non-Lignocellulose and Lignocellulose Materials through Torrefaction

**DOI:** 10.3390/ma14082072

**Published:** 2021-04-20

**Authors:** Nwokolo Nwabunwanne, Tonga Vuyokazi, Adeniji Olagoke, Ojemaye Mike, Mukumba Patrick, Okoh Anthony

**Affiliations:** 1 Department of Physics, University of Fort Hare, P/Bag X1314, Alice 5700, South Africa; 200506777@ufh.ac.za (T.V.); PMukumba@ufh.ac.za (M.P.); 2 SAMRC Microbial Water Quality Monitoring Center, University of Fort Hare, P/Bag X1314, Alice 5700, South Africa; aadeniji@ufh.ac.za (A.O.); mojemaye@ufh.ac.za (O.M.); AOkoh@ufh.ac.za (O.A.); 3 Department of Chemistry, University of Fort Hare, P/Bag X1314, Alice 5700, South Africa; 4 Applied and Environmental Microbiology Research Group (AEMREG), Department of Biochemistry and Microbiology, University of Fort Hare, Alice 5700, South Africa

**Keywords:** biomass, torrefaction, sewage sludge, moisture content, sugarcane bagasse, temperature

## Abstract

Biomass torrefaction is a thermal pre-treatment technique that improves solid fuel properties in relation to its efficient utilization for energy generation. In this study, the torrefaction performance of sewage sludge, a non-lignocellulose biomass and sugarcane bagasse, a lignocellulose biomass were investigated in an electric muffle furnace. The influence of torrefaction temperature on the physiochemical properties of the produced biomaterial were examined. Characterization of the raw and torrefied biomass material were studied using thermogravimetric analysis, Fourier transform infrared spectroscopy (FTIR) analysis and scanning electron microscopy. From the result obtained, it was evident that an increase in torrefaction temperature up to 350 °C caused a 33.89% and 45.94% decrease in volatile matter content of sewage sludge and sugarcane bagasse, respectively. At a higher temperature of 350 °C, the peak corresponding to OH stretching of hydroxyl group decreased in intensity for both biomasses, showing a decomposition of the hydroxyl group as a result of torrefaction. This enriched the lignin content of the torrefied samples, thus making these solid fuels good feedstock for energy production.

## 1. Introduction

Growing interest in the use of biomass material is backed by its cost-effectiveness, sustainability and availability. Biomass materials have the potential to either replace or augment petroleum-derived feedstocks for energy production, as well as in the development of a range of value-added products. For instance, it can be applied in the production of carbon material used in energy storage devices such as supercapacitors [1]. Lignocellulose, representing the most abundant renewable and naturally occurring biomass, comprises of components such as cellulose, hemicellulose and lignin. These components, individually, are important biomaterials with significant applications. Lignin, comprising of 15–30 wt.% of biomass cell wall, can be a promising source of aromatic compounds and chemicals when degraded. It can be used in the production of vanillin, quinones, aromatic acid, vanillic acid and aromatic aldehydes through oxidation reaction [2]. Furthermore, valuable polymers such as polyurethane used in building construction, biomedical applications and electronic products are offshoots of lignin synthesis [3]. Cellulose has also found application in the production of high-performance materials such as fibers, hydrogels, films aerogels and composites. These cellulose-based materials have been fabricated through the use of ionic-based liquid as solvents or additives [4]. In a nutshell, many techniques abound for production of biomaterials with enhanced properties from lignocellulose and non-lignocellulose biomass. A typical example is torrefaction, a thermal pre-treatment technique used in the production of carbon-rich materials called biochar [5]. The produced biochar is an important feedstock for energy production due to improved fuel properties. Utilization of raw biomass as energy feedstock is faced with some challenges, such as high moisture content, low calorific value, high oxygen content, heterogeneous properties and hygroscopic nature [6,7]. These challenges can be overcome through torrefaction. Torrefaction, being a thermal process, makes temperature an important factor of consideration as well as time. The sole goal of torrefaction is to upgrade the inherent properties of biomass materials, including lignocellulose and non-lignocellulose types.

Sugarcane bagasse, a lignocellulose biomass produced as a by-product of sugar milling industries, has a substantial potential for energy production. On an average scale, about 3.3 million tons of raw sugarcane bagasse is generated in South Africa per annum, thus making it an abundant biomass resource within the country [8]. Generally, one ton of sugarcane yields about 300 kg of bagasse, 115 kg of sucrose sugar, 30–50 kg of molasses, 30–40 kg of filter-cake, 3.5 kg of furnace ash and other materials. Sugarcane bagasse composition plays a vital role in its utilization as a feedstock for gasification. Its high moisture content of about 46–52%, low bulk density of about 80–120 kg/m^3^ and fibrous nature can cause agglomeration and de-fluidization during gasification. This will consequently cause a decrease in the yield and quality of syngas produced [9]. Hence, a thermal pre-treatment technique such as torrefaction is required to generate a value-added product from sugarcane bagasse.

Sewage sludge (SS), another viable biomass resource for energy production, usually results from municipal wastewater treatment. It consists of non-toxic organic compounds, a substantial amount of inorganic material, toxic components and high moisture content of about 55–80% [10]. These compositions depend on the SS origin as well as the applied wastewater treatment method. Different SS treatment methods exist, some of which include landfilling, agriculture applications or landscaping, and incineration, anaerobic digestion, gasification, pyrolysis, high-temperature hydrolysis, supercritical oxidation, hydrogen production and production of ethanol and acetone [11,12]. These SS management methods differ by countries, for instance, in South Africa, anaerobic digestion is employed mostly as well as compositing [13]. Agricultural application is often used, although it poses some environmental risks. Incineration, mostly employed in countries with high gross domestic product (GDP), is capital-intensive [11]. Hence, a pre-treatment mechanism such as torrefaction that can create a value-added energy product from sewage sludge waste is needed. Torrefaction of various lignocellulosic materials, such as sugarcane bagasse, woody biomass, cotton stalk, prosopis, barley straw, rice husk, oil palm fiber, sawdust, coffee waste, palm kernel shell as well as non-lignocellulosic sewage sludge, has been studied [11,14,15,16,17,18,19]. However, none have compared the influence of torrefaction temperature on the properties of sugarcane bagasse and sewage sludge to determine their suitability for energy generation. Therefore, this study is focused on the effect of torrefaction temperature on the properties of sewage sludge and sugarcane bagasse. Different torrefaction temperatures ranging from 200 to 350 °C were tested in an electric muffle furnace. Sewage sludge and bagasse were combined in this study for comparison purposes with the aim of determining the possibility of blending the two materials for energy generation through thermochemical conversion.

## 2. Materials and Methods

### 2.1. Fuel Preparation

Sewage sludge, a non-lignocellulose waste, used in this study was sourced from a wastewater treatment plant situated in Alice, Eastern Cape, South Africa. After collection, the sewage sludge sample was air-dried for several weeks before undergoing an oven drying process at a temperature of 105 °C. The moisture content of the raw sewage sludge as-received and prior to air-drying was approximately 80%. On the other hand, sugarcane bagasse, a lignocellulose waste, was received in a dry state and was ground into powder to obtain a homogenous sample of about 600 µm particle size for torrefaction purposes and further characterization.

### 2.2. Torrefaction Process

The torrefaction of the sewage sludge and sugarcane bagasse samples was carried out in an electric muffle furnace with dimensions of 0.49, 0.66 and 0.42 m for length, height and diameter, respectively. Prior to switching on the electric resistance element, the furnace was flushed with nitrogen at 2 L/min until oxygen concentration was less than 1%. The samples (20 g each) were heated from 20 to 200, 250, 300 and 350 °C at a constant heating rate of 10 °C/min for a residence time of 50 min, as obtainable in the literature [11]. An inert atmosphere was maintained in the furnace with the supply of nitrogen gas as well as in controlling the rapid rise in temperature. As the samples in the furnace attained the desired temperature and residence time, the experiment was halted, and the samples were removed immediately. Afterwards, torrefied samples were stored in airtight containers for further analysis.

### 2.3. Thermogravimetric Analysis (TGA)

Thermal degradation characteristics of the torrefied sewage sludge and sugarcane bagasse were studied using Perkin-Elmer TGA 7 (Norwalk, CT, USA). The thermogravimetric analysis (TGA) was carried out at a steady heating rate of 25 °C/min and a 20 mL/min flow of nitrogen. For each thermal analysis (TG), torrefied samples weighing 2.5 mg were placed in the crucible and heated from a temperature of 30 to 900 °C. The samples’ weight loss with respect to temperature and time were recorded for obtaining TG plots. Proximate analysis parameters such as moisture content, volatile matter and ash content of the sample were determined using the modified version of the ASTM D 5142-04 method [8]. All experimental analyses were repeated three times to ensure quality and repeatability of results obtained.

### 2.4. FTIR Analysis

A Fourier transform infrared spectroscopic analysis was conducted to examine the changes in the functional group of raw and torrefied sewage sludge and sugarcane bagasse solid fuels as well as their reactive components. About 3 mg of each solid fuel sample was placed in the sample compartment of an ATR Perkin Elmer 2000 FTIR system for analysis. The spectroscopy then produces an infrared irradiation beam which passes through the samples’ compartment. As the beam passes through, specific energy frequencies are absorbed by the samples, which are depicted in an infrared (IR) spectrum. The produced absorption spectrum shows the samples’ unique characteristics due their constituent molecular functional groups.

### 2.5. Scanning Electron Microscopy Analysis

The micro- and macro-structure of raw and torrefied sewage sludge and sugarcane bagasse were examined using scanning electron microscopy (JSM-6390LV, SEM) (JEOL, Austin, TX, USA). Prior to the analysis and mounting of the sample on a stub of metal, the samples were coated with a thin layer of material, specifically gold, for high-resolution electron imaging. The SEM examinations were performed in a high vacuum mode using an accelerating voltage of 15.0 kV. The high-energy electrons from the SEM then interact with different atoms in the sample to produce various signals that reveal information on the surface morphology of the examined samples.

## 3. Results

In this section, the effects of temperature on thermal properties and molecular composition of torrefied sewage sludge, a non-lignocellulosic material, and sugarcane bagasse, a lignocellulosic biomass are analyzed and discussed. A comparison between the thermal degradation properties, and molecular composition of torrefied sewage sludge and sugarcane bagasse at various temperature is presented.

### 3.1. Thermogravimetric Characterization

**Figure 1:**
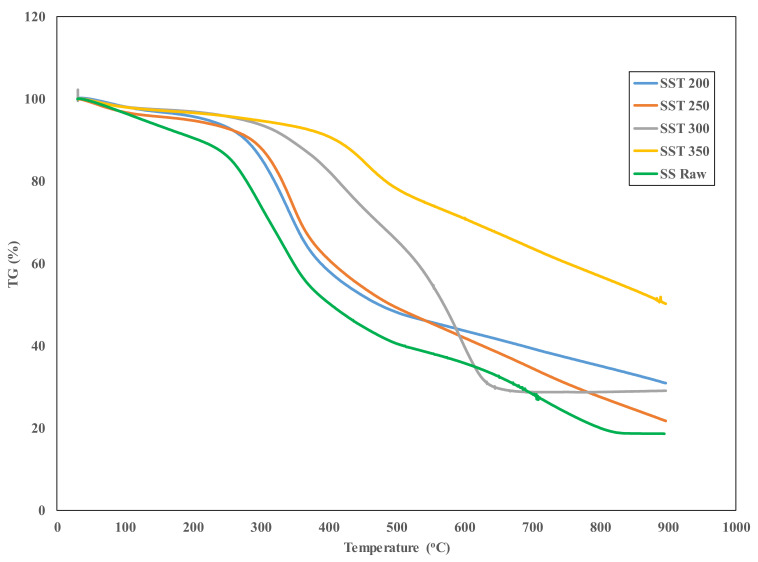
TG curves for raw and torrefied sewage sludge at heating 25 °C/min (SST 200—sewage sludge torrefied at 200 °C, SST 250—sewage sludge torrefied at 250 °C, SST 300—sewage sludge torrefied at 300 °C, SST 350—sewage sludge torrefied at 350 °C, and SS Raw—raw sewage sludge).

**Figure 2:**
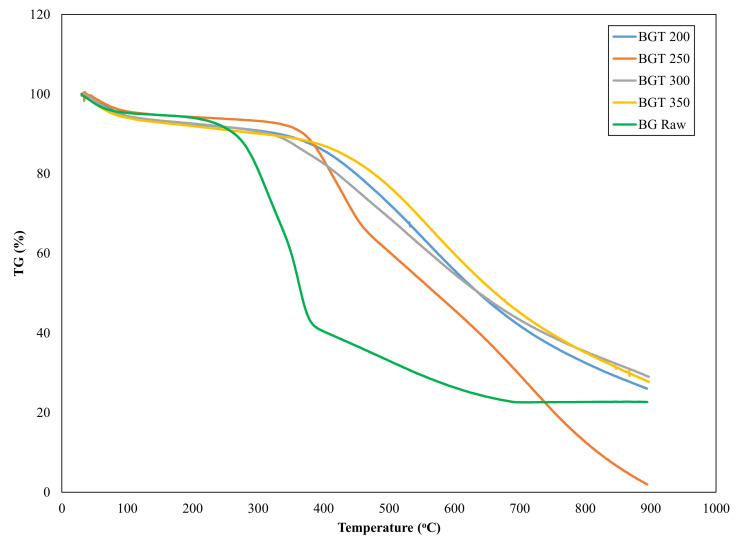
TG curves for raw and torrefied sugarcane bagasse at heating 25 °C/min (BGT200—bagasse torrefied at 200 °C, BGT250—bagasse torrefied at 250 °C, BGT300—bagasse torrefied at 300 °C, BGT350—bagasse torrefied at 350 °C, and BG Raw—raw bagasse).

Thermogravimetric analysis of solid fuels and biomass material in general provides information on the thermal stability of a material as well as the parameters that may influence the thermal degradation characteristics of such biomass. In this analysis, the weight of the biomass samples was measured over time as the temperature changed. The thermogravimetric (TG) curves for raw and torrefied sewage sludge and sugarcane illustrating their thermal transition are shown in Figure 1 and Figure 2.

Under nitrogen atmosphere, the TGA of raw and torrefied sewage sludge in Figure 1 shows steps of degradation over the examined temperature range of 30–900 °C. As observed, the raw sewage sludge (SSRaw) showed four degradation steps, while the torrefied samples showed three-step degradation, as similarly obtained in the pyrolysis process of paper sludge [20]. The first degradation step in raw sewage sludge represented from 30.18 to 205.15 °C, with a percentage weight loss of 9.8%, corresponding to the release of moisture as well as light volatiles. In the case of torrefied sewage sludge samples, the expulsion of moisture and some volatiles occurred from 30.64 to 212.53 °C for SST200 (sewage sludge torrefied at 200 °C) and from 30.45 to 214.32 °C for SST300 (sewage sludge torrefied at 300 °C). Similarly, in Karki et al.’s study [21] on thermal pre-treatment of sewage sludge, the initial weight loss due to loss of moisture was observed before 200 °C. Additionally, the release of moisture in SST200 and SST300 caused a percentage weight loss of 4.72% and 3.57%, respectively. As observed, the sewage sludge sample torrefied at maximum temperatures of 300 °C and 350 °C gave the least weight loss compared to SSRaw and those torrefied at temperatures of 200 and 250 °C. This is associated with more removal of moisture during torrefaction at these temperatures. Evidently, this removal of moisture results in a reduced mass yield as well as an increase in density and grindability of the torrefied sewage sludge [15,22]. In the second degradation step showing the active pyrolysis and oxidation, the temperature spanned from 235.39 to 379.92 °C for raw sewage sludge, with a total weight loss of 34.71%. Whereas, in torrefied sewage sludge, the active pyrolysis and oxidation stage were observed between 273.08 and 438.35 °C for SST200, 286.43–429.71 °C for SST250, 315.50–495.12 °C for SST300 and 562.27–889.21 °C for SST350. Their total weight losses within these temperature ranges are 37.37%, 33.34%, 26.21% and 25.29% for SST200, SST250, SST300 and SST350, respectively. These weight losses at this second stage correspond to the release of volatiles and biodegradable organics such as alcohols and hydrocarbons [20]. For the third step, which marks the final step for all torrefied sewage sludge, the total weight losses of 11.75%, 14.48%, 22.65%, 20.81% and 25.29% were associated with SSRaw, SST200, SST250, SST300 and SST350, respectively. Nevertheless, the SSRaw showed a fourth degradation step with a total weight loss of 18.27%. In the final degradation phase of raw sewage sludge, the sample weight remained almost unchanged from 823.93 to 896.64 °C, similar to sewage sludge torrefied at 300 (SST300). However, the constant sample weight observed in SST300 started at a much lower temperature of 655.34 °C compared to the raw sewage sludge sample. This weight loss is due to the release of carbon dioxide (CO_2_), methane (CH_4_), hydrogen (H_2_) as well as hydrocarbons [23]. The ash contents obtained for the SSRaw, SST200, SST250, SST300 and SST350 at a temperature of 896.64 °C were 18.64%, 30.94%, 21.75%, 29.08% and 50.19%, respectively. As seen, torrefaction caused an increase in the ash content of sewage sludge. For instance, the ash content of sewage sludge torrefied at 350 °C is about 32% greater than the raw sewage sludge. Similarly, in a recent study that evaluated the kinetics of sewage sludge torrefaction, an increase in ash content of 33.1% was reported for sewage sludge torrefied at 300 °C compared to raw sewage sludge [11]. Increase in ash content could be detrimental during gasification of sewage sludge depending on the ash composition. Ash usually contains calcium and iron which, when subjected to high-temperature treatment as is the case of gasification, will cause ash fusion, resulting in slag formation and deposition [24]. This will in turn increase the gasifier system rate of wearing as well as the operating cost. Hence, lower torrefaction temperature is recommended for sewage sludge pre-treatment. Figure 2 presents the TGA plot for raw and torrefied sugarcane bagasse.

From Figure 2, it can be deduced that the thermal degradation profiles for raw bagasse (BGRaw) and bagasse torrefied at 250 °C (BGT250) also showed four distinct stages of degradation, although at different temperature ranges. In contrast, BGT200, BGT300 and BGT350 showed three weight loss stages, drying stage, devolatilization stage and constant rate stage. All the solid fuels had the drying phase (30–145 °C), which resulted in a weight loss of 6.10% for BGRaw, 5.96% for BGT200, 5.68% for BGT250, 5.47% for BGT300 and 5.76% for BGT350. Previous studies have shown that vaporization of most biomass material occurs at a temperature range of 25–150 °C, as was the case for some waste biomass residue, such as cashew pruning, mango pruning, flower stems, Husk of babssu coconut and banana pseudostem [23]. The drying phase for the raw and torrefied bagasse occurred within five minutes and it signifies evaporation of moisture and some light volatiles. A slight decrease in moisture content observed within this first phase with an increase in temperature revealed that torrefaction affected the hydrophobicity of bagasse. Probably, this could be due to the loss of hydroxyl group originating from degradation of hemicellulose content [25]. Most notably, devolatilization of BGRaw became significant at a lower temperature of 260 °C compared to torrefied bagasse samples that began at higher temperature of 305 °C for BGT250 and 400 °C for other torrefied temperatures. This simply implied that torrefaction increases the thermal stability of the samples, thus making it more resistant to thermal degradation as obtainable in coal solid fuel. Saeed et al. [26] attributed this thermal resistance to increase in energy bond as well as coal polycyclic aromatic structure. The maximum weight loss (devolatilization stage) for BGRaw and BGT250 occurred between 258 and 600 °C because of hemicellulose, cellulose and some percentage of lignin degradation. Recent research shows that the degradation of these organic components causes the release of some gases, such as methane (CH_4_), CO_2_, CO and water vapor [27,28]. We can envision that the release of these gases will cause an increase in the carbon content and a reduction in oxygen content of bagasse. In Carrier et al.’s [19] study, the maximum weight loss for crude wood, washed wood and macro-components extracted from the washed wood were recorded in the range 300–350 °C. Table 1 presents the proximate analysis of raw and torrefied sewage sludge and sugarcane bagasse. A comparison of Figure 1 and Figure 2 showed slight differences in the effect of torrefaction treatments at varying temperature ranges for the organic matter contained in sugarcane bagasse and sewage sludge samples. This could be attributed to the composition of the samples. According to the literature, sewage sludge is composed of microbes, undigested organic matter (paper, plant materials, fecal matter, oils), metal ions, silicates, pesticides, organic macromolecules (hydrocarbons), proteins and water [12,29,30], while sugarcane bagasse contains cellulose, lignin, proteins, crude fibers, ash and fat [31] in various proportions. Although, we did not analyze the proportion of these various components. However, a closer look at the proximate analysis in Table 1 shows that the difference between the volatile matter contents between sugarcane bagasse and sewage sludge is only 8.94% in the raw samples. Therefore, this could be the rationale behind the slight variation among the two samples.

Table 1 lists the proximate analysis in weight percentage of raw and torrefied sewage sludge and sugarcane bagasse samples. Torrefaction at different temperatures strongly influenced the properties of sewage sludge and sugarcane bagasse, as depicted. Volatile matter (VM), a quantitative measure of solid fuel reactivity, showed a clear decreasing trend with increase in torrefaction temperature for sewage sludge and sugarcane bagasse samples. The volatile matter of raw sewage sludge decreased from 53.09% to 19.20% for sewage sludge torrefied at 350 °C, while raw bagasse decreased from 62.03% to 16.09% for the sample torrefied at 350 °C. Comparatively, Pimchuai et al.’s [32] study showed a decrease in VM content of approximately 26.87% and 28.01% for raw bagasse and water hyacinth torrefied at a temperature of 300 °C. Apparently, this trend of decrease in volatile matter content along with the increase in torrefaction temperature is similar in other recent studies [9,33,34,35]. The observed decrease in VM content of sewage sludge is due to decomposition of protein, lipids, carbohydrates and other organic matter present, while the decrease in bagasse VM is attributed to depolymerization of biomass polymers, particularly hemicellulose and the release of volatiles during torrefaction [23,36,37]. For FC content of sewage sludge, there was a step backward and forward in the percentage composition owning to its complex chemical composition characterized by the presence of organic and inorganic materials in the sewage sludge. Firstly, there was a 3.71% decrease as raw sewage sludge was torrefied at 200 °C, which was followed by an increase of 8.60%, 9.63% and 8.54% for samples torrefied at 250, 300 and 350 °C, respectively. Contrarily, sugarcane bagasse showed a clear increasing trend in FC percentage composition as torrefaction temperature increased. The observed increase in FC content of sugarcane bagasse alongside a decrease in VM content is advantageous in that it enhances the energy value of the biomass [21,38]. On a similar note, Kanwal et al.’s [25] study reported an increase in FC of sugarcane bagasse from 16.09% to 42.79% as torrefaction temperature rose from 200 to 300 °C. Increase in FC is beneficial during gasification, as a higher percentage of FC will lead to greater heat of combustion as well as enhanced calorific value of syngas produced. In terms of ash content, sugarcane bagasse torrefied at a temperature of 250 °C gave the least percentage composition of 1.75%. As the torrefaction temperature rose to 350 °C, an increase in ash content of 28.64% and 25.84% was observed for sewage sludge and sugarcane bagasse samples, respectively. Previous studies explained this increase in ash content to be attributed to loss of organic matter content during torrefaction as well as accumulation of remnants of non-decomposed inorganic compounds [39,40]. High ash content in sewage sludge torrefied at 350 °C can be disadvantageous in its use as an energy source due to slagging, agglomeration and fouling problems associated with it, particularly during gasification [24]. The pre-treatment of sewage sludge and sugarcane bagasse through torrefaction lowered the equilibrium moisture content, which indicates an increase in hydrophobicity of the biomass materials, as evidenced in Table 1. The hydrophobicity nature of the biomass materials lowered their moisture adsorption rate, and gasification of such biomass will increase the heat released at the oxidation zone of the gasifier system. Hence, the unconverted tars and gases that descend from the pyrolysis zone into the oxidation zone will be easily combusted, leading to a syngas with low tar content.

### 3.2. Fourier Transform Infrared Spectroscopy (FTIR) Analysis

**Figure 3:**
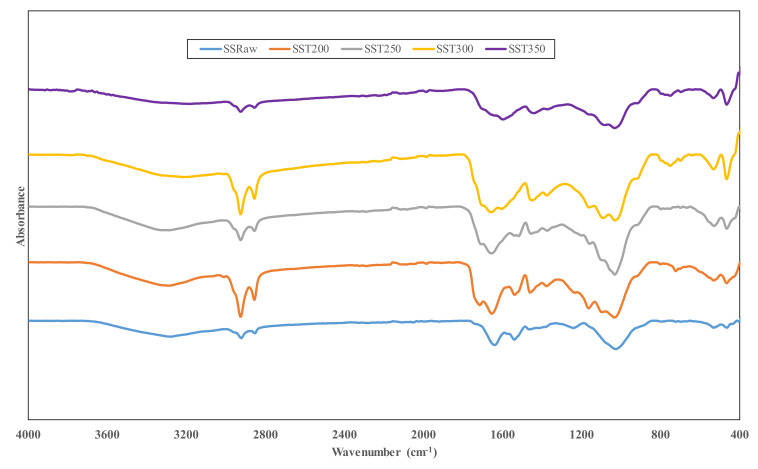
FTIR spectra of raw and torrefied sewage sludge at varying temperature.

**Figure 4:**
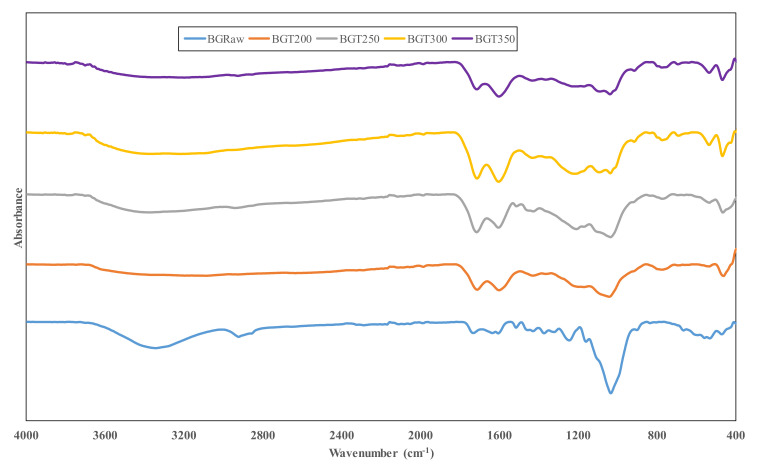
FTIR spectra of raw and torrefied sugarcane bagasse at varying temperature.

Generally, FTIR analysis assists in the determination of a biomass molecular composition and structure by measuring the amount of infrared light absorbed by the biomass sample at various wavelengths. In the present study, structural changes in torrefied sewage sludge and sugarcane bagasse were measured in the infrared mid-range IR of 4000 to 400 cm^−1^. Figure 3 and Figure 4 present the FTIR spectra of sewage sludge and sugarcane bagasse in raw and torrefied state.

The vibrational peaks across the sewage sludge spectra shown in Figure 3 reveal the presence of various functional groups peculiar to the following compounds, such as phenol (C–O–C), alkanes (C–H), alkenes (C=H), alcohol (C–OH), nitrogenous compounds (N=H), ketones (C=O), ester and aromatic/heterocyclic compounds, as well as moisture (H–O–H). The bands in the infrared spectrum assigned to both the torrefied and raw sewage sludge were done following the characterized FTIR presented in the literature [14,35,41,42,43,44]. The variation in the spectra reading for the torrefied and raw sewage sludge were as a result of the degradation of the organic polymers present in the samples after the torrefaction treatments. An intense peak occurring at 1026 cm^−1^ is linked to OH vibrations of mineral compounds in the sewage sludge. Vibrations observed between 3400 and 3000 cm^−1^ for raw sewage are due to OH stretching of phenol, hydroxyl group and alcohol, as well as NH stretching of amines and amide groups. This peak slightly decreased in intensity with higher torrefaction temperature, particularly at 350 °C, suggesting the decomposition of hydroxyl groups during torrefaction. In all the sewage sludge samples, the double vibrational peak observed within the wavenumber region of 2950–2800 cm^−1^ is a typical characteristic of the hemicellulose spectrum [45]. It indicates the presence of alkanes and corresponds to aliphatic C–H bond vibrations in CH_2_ and CH_3_ groups. The double vibrational peak first increased in intensity for the sample torrefied at 200 and 300 °C, and later decreased in intensity for sewage sludge torrefied at 350 °C, showing the decomposition of organic fatty hydrocarbons to form methane, carbon dioxide and aromatic structure [46]. The peak observed between 1720 and 1620 cm^−1^ indicates the presence of ketones, esters and aldehydes in sewage sludge samples. The oxidation of ketones usually results in the formation of a desirable biofuel compound known as ester [47]. A small vibration seen between 1600 and 1450 cm^−1^ is associated with –NO_2_ stretching and NH bending, and this signifies the presence of nitrogen compounds. Nitrogen compounds usually result from dehydration reactions of peptide bonds emanating from protein fraction of the sewage sludge [48]. The presence of carboxylic group, according to a previous study, is said to influence the thermal strength of a biomass due to hydrogen interaction [49]. Figure 4 presents the infrared spectra of raw sugarcane bagasse torrefied at various temperatures. 

Vibrational bands shown in Figure 4 correspond to the functional group characteristic of bagasse samples during their exposure to the infrared mid-region of the electromagnetic spectrum. The bands in the infrared spectrum were assigned to the bagasse functional groups, as contained in the literature described above. The vibration band at a wavenumber of 3342 cm^−1^ corresponding to O–H bond was obvious in the raw bagasse. However, the O–H band intensity decreased within the wavenumber range of 3400–3000 cm^−1^ in the torrefied samples, indicating a loss of this functional group. This is attributed to dehydration reactions that occur during torrefaction, which in turn improved hydrophobicity of torrefied bagasse. The presence of this band in the raw bagasse is due to the involvement of hydroxyl, alcoholic and phenolic groups in hydrogen bonding, particularly in carbohydrates and lignin [41,50]. A small peak at 2920 cm^−1^ for raw bagasse corresponds to asymmetric C–H bonds stretching of aliphatic hydrocarbon within methyl and methylene groups of cellulose. Notably, this peak became less intense in the torrefied bagasse samples. This is consistent with the report that aromatic and aliphatic hydrocarbon decrease with increase in temperature, due to the conversion of weak hydrogen bonds to more stable ones [51]. Absorption bands that occurred at about 1730–1710 cm^−1^ for both torrefied and raw bagasse samples are associated with stretching vibrations of C=O groups. These vibrations are traceable to the presence of carboxylic acids such as xyloglucan, galactoglucomannan and arabinoglucuronoxylan in hemicellulose [7]. The peak noted at wavenumber 1633 cm^−1^ for raw bagasse corresponds to C=C stretching of the aromatic ring in plane vibration. Upon torrefaction, this peak increases in intensity and moves to a lower wavenumber of approximately 1600 cm^−1^ due to the formation of non-polar and unsaturated compounds through degradation of hemicellulose [52]. This in turn enriches the lignin content of the torrefied bagasse, thus making it a good feedstock for gasification and combustion. The existence of a double peak in all samples’ spectra within the region of 1800−1500 cm^−1^ is a characteristic of the lignin IR spectrum [30,53]. Furthermore, the strong vibrational band in the raw bagasse observed at a wavenumber of 1032 cm^−1^ is due to vibrations in C–O, C=C and C–C–O in cellulose, hemicellulose and lignin bio-polymer components. However, this band decreased in intensity with torrefied bagasse samples and occurred at wavenumbers of 1040 cm^−1^ for BGT200, 1034 cm^−1^ for BGT250, 1090 cm^−1^ for BGT300 and 1036 cm^−1^ for BGT350. Hence, this indicates that raw bagasse contained higher C–OH (ethanol) compound compared to torrefied samples.

### 3.3. Morphological Characterization

**Figure 5:**
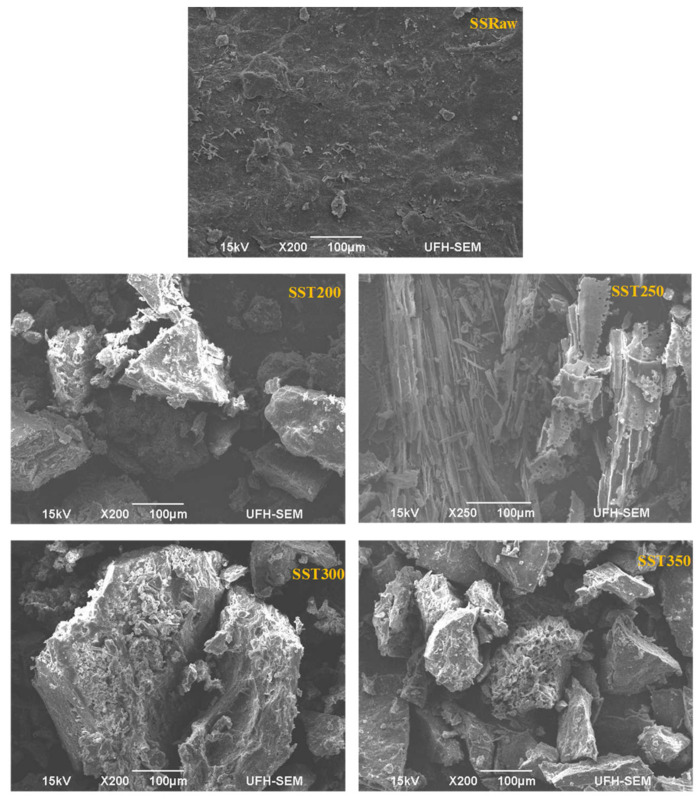
SEM images of raw sewage sludge (SSRaw) and torrefied sewage sludge at 200 °C (SST200), 250 °C (SST250), 300 °C (SST300) and 350 °C (SST350).

**Figure 6:**
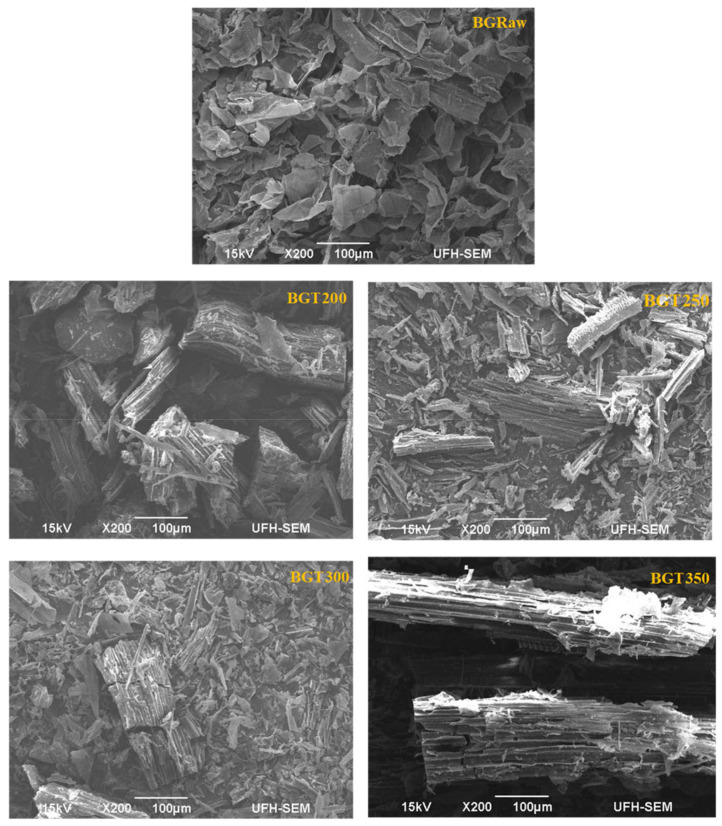
SEM images of raw bagasse (BGRaw) and torrefied bagasse at 200 °C (BGT200), 250 °C (BGT250), 300 °C (BGT300) and 350 °C (BGT350).

Scanning electron microscopy (SEM) images of sewage sludge and sugarcane bagasse in their raw state and when torrefied at different temperatures are presented in Figure 5 and Figure 6.

The torrefaction process alters the morphological structure of any biomass material, which brings the need for SEM analysis. In addition, the degradation of organic matter during torrefaction can be easily observed through SEM analysis. The SEM images in Figure 5 reveal that raw sewage sludge (SSRaw) is characterized by spherically shaped particles of varying sizes. Evidently, torrefaction had an impact on the surface topology of the sewage sludge, as observed. At a temperature of 200 °C, represented by SST200, the spherical-shaped particles experienced some level of degradation and started fusing into an irregular shaped, medium-sized lump. Further degradation of the particles into a coherent structure with small openings was observed at a temperature of 250 °C (SST250). These openings were created because of DE volatilization of the sewage sludge, which gave way for escape of gases. During torrefaction at 300 °C, the disintegrated particles aggregated into larger spongy lumps with more visible openings. At torrefaction temperature of 350 °C, denoted by SST350, the lumps were broken into rock-like structures.

The surface topology of the raw bagasse sample (BGRaw) displayed in Figure 6 shows a flake-like structure consisting of fibers and pitch. Upon torrefaction at a temperature of 200 °C, the existing structures are ruptured due to the thermal pre-treatment. A new tread-like structure is formed from the degraded particles, as observed in BGT200. An increase in temperature to 250 and 300 °C resulted in a further disintegration of the constituent particles into a granular fragmented structure, as seen in BGT250 and BGT300. Although the disintegration of particles was similar in BGT250 and BGT300, their particle sizes differed, and BGT300 had smaller and more dispersed particles compared to BGT250. The reduced particle sizes can be associated to decomposition of hemicellulose, cellulose and lignin composition of bagasse [25]. As the temperature increased to 350 °C, the tiny dispersed particles agglomerated into a tubular-shaped structure with voids. These voids were created as pathways for the gas products released during torrefaction. Notably, the agglomeration of the particles is attributed to the binding effect of degraded lignin on plant cell walls due to the presence of agglomerates such as silicon (Si) and phosphorus (P). Previous studies noted that this binding nature of lignin is what enhances the grindability of torrefied solid fuels [54,55].

Besides the use of torrefied sewage sludge and sugarcane bagasse (biochar) as a feedstock for energy production, it can equally serve as a soil conditioner. Previous studies have enumerated some proposed benefits of applying biochar to soil. These include soil pH enhancement, reduction in soil nutrient leaching and heavy metal availability, increase in plant nutrient availability and use, improvement in cation exchange capacity and water holding capacity. Achievement of these benefits is hinged on the characteristics of the produced biochar. Biochar characteristics are influenced by a number of factors, some of which include biomass type and properties, treatment temperature and residence time. Among these parameters, temperature has been found to have the most significant effect [56,57]. Thus, a proper standardized method needs to be adopted when converting biomass to biochar.

## 4. Conclusions

Torrefaction performance and characteristics of sewage sludge and sugarcane bagasse samples have been investigated in the present study. Evidently, an enhancement in the properties of the torrefied samples were observed with the increase in temperature. For instance, the thermogravimetric analysis showed that the devolatilization phase for raw sewage sludge and sugarcane bagasse commenced at temperatures of 235 and 260 °C respectively, while for the torrefied samples, this was initiated at higher temperatures that ranged from 273 to 562 °C, with SST350 and BGT350 showing the highest initiation temperature. This implied that the increase in torrefaction temperature led to a corresponding increase in the thermal stability of the samples. Thus, making the biomass more resistant to thermal degradation as obtainable in coal solid fuel. In terms of ash yield, BGT250 performed optimally with the lowest ash content, while SST350 had the highest ash yield. Noting that high ash content is undesirable, the study recommends that sewage sludge torrefaction temperature should not exceed 300 °C. In addition, the surface topology of the samples as examined through scanning electron microscope showed an agglomeration of disintegrated particles at higher temperature, which equated to better grindability of the torrefied materials.

## Figures and Tables

**Table 1:** Proximate analysis of raw and torrefied sewage sludge and sugarcane bagasse.

Sample	Proximate Analysis (wt.%)			
	MC	VM	AC	FC
SSRaw	8.39	53.09	18.51	20.01
SST200	4.30	48.51	30.89	16.30
SST250	3.23	46.52	21.64	28.61
SST300	2.67	38.52	29.17	29.64
SST350	1.97	19.20	50.28	28.55
BGRaw	6.55	62.03	22.36	9.06
BGT200	5.90	56.20	26.21	11.69
BGT250	5.26	51.7	1.95	40.65
BGT300	4.71	24.18	28.88	42.23
BGT350	4.23	16.09	27.59	52.09

MC—moisture content, VM—volatile matter, AC—ash content, FC—fixed carbon.

## Data Availability

Data will be made available on request as some of the findings are yet to be published.
